# Neurological Complications in Active Left-Sided Infective Endocarditis Requiring Early Surgery

**DOI:** 10.3389/fcvm.2021.716233

**Published:** 2021-12-03

**Authors:** Yolanda Carrascal, Bárbara Segura, Eduardo Velasco, Ángel L. Guerrero

**Affiliations:** ^1^Cardiac Surgery Department, University Hospital of Valladolid, Valladolid, Spain; ^2^Department of Surgery and Medicine, University of Valladolid, Valladolid, Spain; ^3^Neurology Department, University Hospital of Valladolid, Valladolid, Spain

**Keywords:** endocarditis, cardiac surgery, neurology, neurological complications in cardiac surgery, urgent surgery

## Abstract

**Introduction:** To determine whether preoperative symptomatic neurological complication (SNC) predicts a worse prognosis of patients with active left-sided infective endocarditis who required early surgery.

**Methods:** We conducted a retrospective chart review and analyzed risk factors for SNCs and immediate, medium-term, and long-term mortality in patients with active left-sided infective endocarditis who required early surgery (median follow-up: 70.5 months).

**Results:** Of 212 included patients, preoperative SNCs occurred in 22.1%. Independent risk factors for preoperative SNC included early hospital admission (<10 days after symptoms onset), duration of antibiotic therapy <7 days, vegetation diameter > 30 mm, preoperative chronic therapy with steroids, and peripheral embolism. A new postoperative SNC occurred in 12.7% of patients. No significant differences related to preoperative or postoperative SNCs were observed in postoperative mortality (29.8% vs. 31.5%) or during follow-up. No significant differences in postoperative mortality were observed between hemorrhagic or ischemic SNCs. There was a non-significant trend to increased mortality in patients who underwent surgery within 7 days of presenting with SNC (55.5%) compared to those who underwent surgery more than 7 days after SNC (33.3%) (*P* = 0.171). Concomitant risk of mortality or postoperative hemorrhagic transformation increased when surgery is required during the first week after preoperative SNC (77.5% vs. 25%) (*P* = 0.017).

**Conclusions:** Patients with active left-sided infective endocarditis who need early hospital admission are at a higher risk of SNC. Mortality is higher in patients who underwent surgery within 7 days of SNC, but mortality of early surgery is acceptable after the first week of preoperative ischemic or hemorrhagic complication. We have not been able to demonstrate that preoperative nor postoperative SNCs predicted a reduced immediate, medium-term, or long-term survival in the population analyzed in this study.

## Introduction

The incidence of infective endocarditis (IE) has increased from 2.7 to 3.4 per 100,000 patients/year in Spain during the last decade (1). IE mortality, which is around 20%, has not changed in recent 15 years but is higher in patients who require early surgical treatment (2, 3) and also when associated with a preoperative symptomatic neurological complication (SNC) such as stroke (4–7). The global incidence of cerebral embolism secondary to IE ranges between 20 and 50%, although the Spanish National Health System reported only 9% between 2003 and 2014 (1). Only one recent publication mentioned neurological complications secondary to IE in Spain: around 50% of patients with IE underwent surgery but this percentage dropped to 32% when preoperative clinical symptoms and imaging evidence of SNC appeared (6). This study aimed to 1. assesses the risk factors of preoperative SNC and 2. determine whether preoperative SNC predicts a worse prognosis of patients with active left-sided infective endocarditis (ALSIE) who require early surgery. Secondary aims included the assessment of other risk factors of mortality in this sample, such as time to surgery. We evaluated the incidence and risk factors for pre- and postoperative SNC, the morbimortality associated with surgical procedures, and the medium- and long-term survival in patients with ALSIE requiring early surgical treatment during the in-hospital stay.

## Materials and Methods

**Figure 1:**
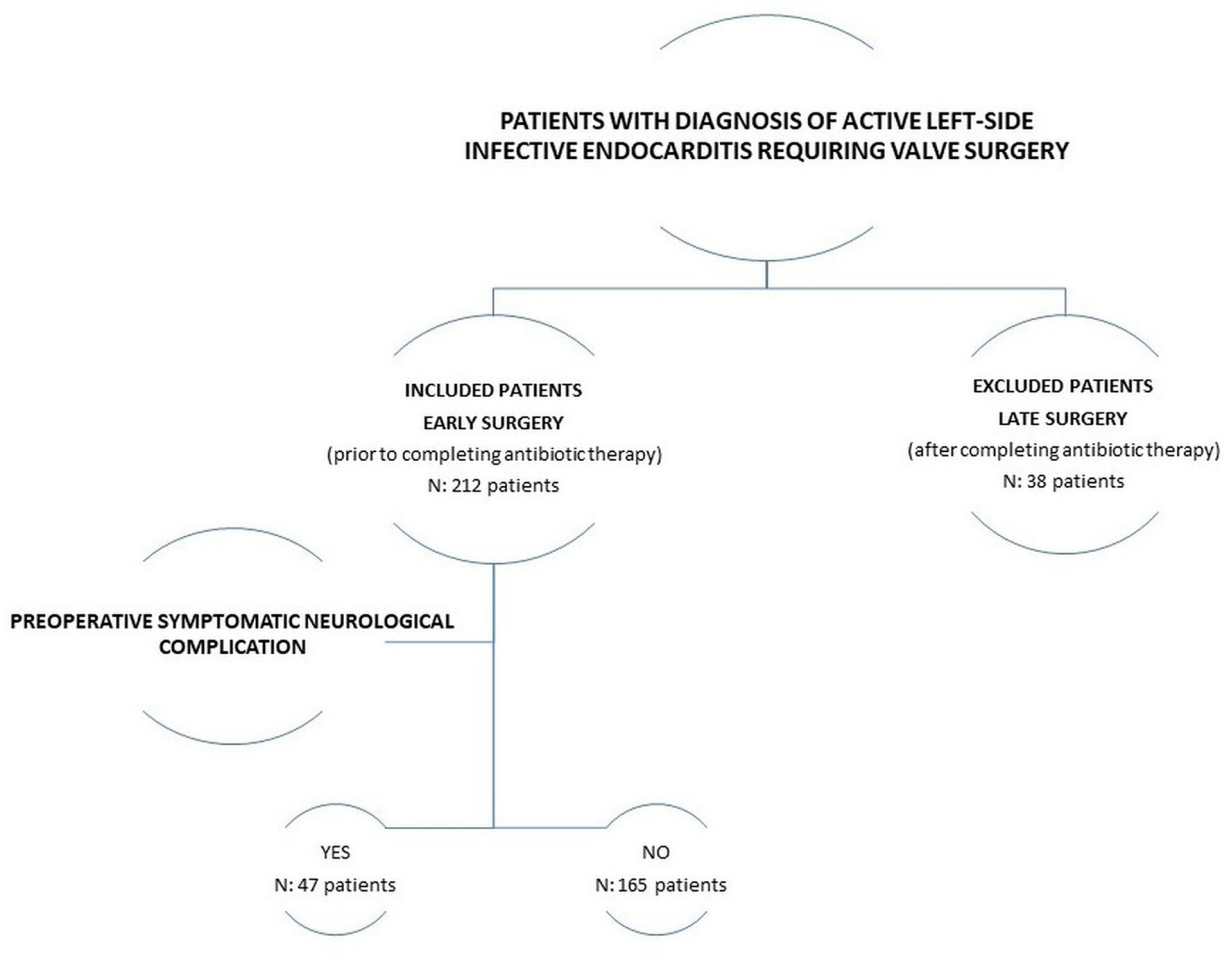
Flow chart of the study design.

This is an analytic observational study with a retrospective cohort design. Participants were included if they were diagnosed with ALSIE and required early surgical treatment. Patients with left-sided valve cured IE, right-sided IE, or cardiac device-related IE were excluded from the study (Figure 1). The study was done according to the strengthening of the reporting of observational studies in epidemiology guidelines (8). The study protocol (identification number PI19-1236) received full approval from both the local Institutional Research Review Committee and the Clinical Research Ethics Committee. Informed consent was obtained from each patient. The study period included January 2000 to May 2020. Prospectively collected demographic data and qualitative and quantitative [hemoglobin (g/dl), hematocrit (%), platelet count (×103/μl), white blood cell count (×103/μl), creatinine (mg/dl), glomerular filtration rate (ml/min/1.73 m^2^), procalcitonin (ng/ml), and protein C reactive (mg/l)] risk factors of patients with ALSIE, who required early surgery in a tertiary academic hospital, were analyzed. These patients were classified into two groups according to the presence of preoperative SNC. The asymptomatic neurological damage related to IE (showed in systematic neuroimaging procedures in patients with IE but not corresponding with neurological clinical symptoms during exploration) does not seem to increase the risk of mortality, and it is not a clear contraindication for early surgery in patients with ALSIE (4). Therefore, we only analyzed patients with preoperative SNC. The microbiological diagnosis was determined from blood cultures and/or heart valve tissue cultures. We conducted a follow-up of surviving patients. Data were prospectively collected during the follow-up. Patients were not involved in clinical research.

### Definitions of the Variables Analyzed

#### Early Surgery

Surgery performed before completing antibiotic therapy. Following the criteria established in the guidelines of the European Society of Cardiology (9), early surgery was performed in patients with heart failure, in patients with uncontrolled local or systemic IE, and to prevent systemic embolic events.

*Operative mortality* was defined as death occurring within the first 30 days of surgery or during the hospital stay if the patient was not discharged before this time.

#### Emergency Surgery

Surgery performed within 24 h after the need for early surgery is determined; *urgent surgery:* within a few days; *elective surgery*: after at least 1–2 weeks of antibiotic therapy.

#### Preoperative SNC

Any neurological injury (classified into ischemic, hemorrhagic, brain abscess, and meningoencephalitis [associated with cerebrospinal fluid culture] according to the predominant lesion) that resulted in clinical symptoms of at least 30 min and was accompanied by a brain lesion detected by at least one neuroimaging study (CT and/or MRI). According to the findings of the neuroimaging studies, the neurological embolic injury was categorized as macroembolic (when focal neurologic damage affected the major cerebral vessel), microembolic, or mixed.

#### Healthcare-Associated IE

When fever appeared >48 h after hospital admission or after an invasive procedure performed within the previous 6 months.

*Early-onset prosthetic valve endocarditis* was a case that occurred within 1 year of valve replacement.

*Time to surgery* was the number of days that elapsed from the onset of clinical IE symptoms to surgery. *Time to admission* was the number of days between the onset of IE symptoms and hospital admission. *Duration of antibiotic treatment* was the number of days of adequate antimicrobial therapy. *Time to neurological event* was the number of days between the onset of IE symptoms and detected SNC.

#### Prosthetic Valve Dysfunction

Paravalvular regurgitation identified by echocardiography, with or without rocking motion of the prosthesis and/or increased transprosthetic gradient due to IE (9).

*Acute kidney injury* was defined according to the risk, injury, failure, loss, and end-stage criteria (10).

The surgical risk was preoperatively estimated according to the European System for Cardiac Operative Risk scales (11).

*Postoperative low cardiac output* was diagnosed when cardiac index was <2.2 l/min/m^2^, diuresis was <0.5 ml/kg/h and ventral venous oxygen saturation was <60%, and/or lactate was >3 mmol/l, in the absence of relative hypovolemia (12).

*Cardiac arrhythmias* were identified by electrocardiographic patterns compatible with fibrillation and atrial flutter and second- or third-degree atrial-ventricular blockages.

*Postoperative SNC* was diagnosed by evidence of a symptomatic embolic, thrombotic or hemorrhagic event of at least 30 min, with or without residual motor, sensory, or cognitive deficit. Stroke was diagnosed when symptoms persisted beyond 24 h; the transient ischemic attack was otherwise diagnosed.

The following were evaluated during follow-up: incidence of reinfection (new episode of IE caused by a different microorganism or by the same but >6 months after the initial episode), relapse (new episode of IE caused by the same microorganism <6 months after the initial episode), new SNC and redo-surgery related to IE.

### Statistical Analyses

Statistical analyses were performed using SPSS software version 22.0. Quantitative variables are expressed as mean (SD), median (interquartile range) for asymmetric distributions, or as hazard ratios (95% CI) when appropriate. The Kolmogorov–Smirnov test was used to determine the normality of the distribution for each variable. Qualitative variables are expressed as counts (percentages). In univariate analyses, hypothesis testing was done using the chi-squared or Fisher's exact tests for qualitative variables (the Bonferroni correction was applied for multiple comparisons) and the Student's *t*-test or Mann–Whitney U tests for quantitative variables. Associations between risk factors, mortality, and preoperative or postoperative neurological complications found to be significant in univariate analyses (*P* < 0.2) were entered stepwise into Cox proportional hazards models. We analyzed mortality risk factors and actuarial event-free survival using a Cox model and the Kaplan–Meier test. A *P* < 0.05 was considered significant. To adjust the Cox models, we considered all preoperative variables found significant (*P* < 0.2) in the univariate analyses.

## Results

### Demographics and Clinical Characteristics

We included 212 consecutive patients with ALSIE who required early surgery. Of these, 53.3% of patients required emergency surgery, the indication being heart failure in 67.5% of the cases. The preoperative characteristics are detailed in Table 1. The primary source of the infection was unknown in 51.9% of the patients, and blood cultures were sterile in 18.4%. Healthcare-associated (20.3%), gastrointestinal (10.4%), and genitourinary (6.2%) sources were the most common sources of primary infection. Forty-seven patients (22.1%) presented with preoperative SNC secondary to IE (Table 2). SNC was the first clinical manifestation of IE in 21 cases (9.9%). No fever or other typical symptoms of IE were referred by the patients before neurological event. The most frequent clinical presentation of SNC was stroke. Operative mortality was 31.1% for all 212 patients (29.8% in patients with preoperative SNC) (*P* = 0.821), and uncontrolled sepsis was the most frequent cause (23 cases). We conducted an in-person follow-up of all 146 surviving patients, with a mean follow-up time of 83.42 ± 64.13 months (median: 70.5 months, range: 1–245 months).

**Table 1:** Demographic profile and preoperative clinical characteristics in patients with active left-sided infective endocarditis, with or without preoperative SNC.

**Variable**	**No SNC**	**SNC**	***P***
	**(*n* = 165)**	**(*n* = 47)**	
**CLINICAL DATA**			
Age (years)	62.6 (13)	61.9 (14.7)	0.717
Female sex	34 (20.6)	17 (36.2)	**0.028**
Peripheral vascular disease	5 (3.0)	6 (12.8)	**0.008**
Other than neurological embolism	42 (25.5)	33 (70.2)	**<0.0001**
Time to admission <10 days	78 (47.2)	32 (68)	**0.012**
Healthcare-associated IE	34 (20.6)	9 (19.1)	0.827
Arterial hypertension	77 (46.7)	24 (51.1)	0.594
Diabetes	36 (21.8)	8 (17.0)	0.474
Dyslipidemia	57 (34.5)	16 (34.0)	0.949
Renal failure	40 (22.5)	7 (20.6)	0.809
Haemolytic anaemia	16 (9.7)	7 (14.9)	0.312
Coronary disease	46 (23)	1 (8.3)	0.235
Time to SNC (days)	–	2 (14)	–
SNC-surgery (days)	–	15 (14)	–
Onset of fever-surgery (days)	36.3 (30)	36.7 (40.3)	0.943
Time to admission (days)	10 (26)	4 (14)	0.576
Time to surgery (days)	30 (28)	24 (27)	0.943
Haemoglobin (g/dL)	10 (4)	10 (4.1)	0.929
**LOCALIZATION**			
PVE	47 (28.5)	20 (42.6)	0.067
Aortic IE	82 (49.7)	22 (46.8)	0.727
Mitral IE	60 (36.4)	19 (40.4)	0.611
Multivalve IE	33 (20)	7 (14.9)	0.430
Early-onset PVE	20 (12.1)	9 (19.1)	0.216
**MICROBIOLOGICAL DIAGNOSIS**			
Blood culture-negative IE	30 (18.2)	9 (19.1)	0.880
*S. aureus*	34 (20.6)	14 (29.8)	0.192
Methicillin-resistant *S. aureus*	13 (7.9)	2 (4.3)	0.530
*S. bovis*	9 (5.4)	3 (6.3)	0.808
*S. agalactiae*	7(4.2)	3 (6.3)	0.541
Other streptococci	30 (18.1)	8 (17)	0.854
Coagulase negative staphylococci	20 (12.1)	4 (8.5)	0.567
**Gram negative bacteria**			
HACEK species	1 (0.6)	0 (0)	0.592
Non-HACEK species	4 (2.4)	1 (2.1)	0.190
Enterococci	24 (14.5)	1 (2.1)	**0.019**
Fungi	2 (1.2)	4 (8.5)	0.074
Aerococcus	1 (0.6)	0 (0)	0.592
Valve culture negative	77 (46.7)	22 (46.8)	0.986
**ECHOCARDIOGRAPHIC FEATURES**			
Vegetations	144 (87.3)	42 (89.4)	0.700
Diameter <10 mm	38 (23)	7 (14.9)	0.229
>20 mm	56 (33.9)	19 (40.4)	0.412
>30 mm	10 (6.1)	8 (17)	**0.017**
Annular abscess/pseudoaneurysm	66 (40)	18 (38.3)	0.833
Prosthetic valve dysfunction	36 (21.8)	16 (34)	0.086
LVEF> 50%	140 (84.8)	42 (89.4)	0.610
**NYHA FUNCTIONAL CLASS**			
I	8 (4.8)	1 (2.1)	
II	26 (15.8)	11 (23.4)	0.568
III	61 (37.0)	17 (36.2)	
IV	70 (42.4)	18 (38.3)	
**PREOPERATIVE TREATMENT**			
Antibiotic treatment before surgery (days)	11 (14)	16 (11)	**0.023**
Antibiotic (>7 days) before surgery/SNC	109 (66.1)	10 (21.3)	**<0.0001**
Intravenous inotropes	53 (32.1)	13 (27.7)	0.560
Anticoagulant	40 (24.2)	18 (38.3)	0.057
Calcium channel blockers	16 (9.7)	1 (2.1)	0.128
Chronic steroid treatment	21 (12.7)	11 (23.4)	0.071
**INDICATIONS FOR EARLY SURGERY**			
Heart failure	119 (72.1)	24 (51.1)	
Uncontrolled infection	39 (23.6)	7 (14.9)	**<0.0001**
Prevention of embolism	7 (4.2)	16 (34)	
**SCORE RISK**			
Logistic EuroSCORE	27.9 (21.3)	41.9 (26.5)	<0.0001
EuroSCORE II	16 (16.3)	21.6 (17.3)	0.041

*Qualitative values are presented as number (%), quantitative values are presented as mean (standard deviation), or median (interquartile range) in asymmetric distributions. SNC, symptomatic neurological complication; PVE, prosthetic valve endocarditis; HACEK, Haemophilus Aggregatibacter Cardiobacterium Eikenella Kingella; LVEF, left ventricular ejection fraction. Bold values statistically significant in univariate analysis (p < 0.05)*.

**Table 2:** Characteristics of patients with preoperative SNC (*n* = 47).

**Variable**	**No. (%)**
Ischemic stroke	33 (70.2)
Hemorrhagic stroke	12 (25.5)
Meningoencephalitis	2 (4.3)
**Cerebral Imaging techniques (CT/MRI)**	
Multiple lesions	23 (48.9)
Macroembolism	32 (68.1)
Microembolism	8 (17.0)
Both	7 (14.9)
**Clinical presentation**	
Stroke	41 (87.2)
Transitory ischemic accident	5 (10.6)
Seizures	1 (2.1)

*SNC, symptomatic neurological complication; CT, computed tomography; MRI, magnetic resonance imaging*.

### Effect of Preoperative SNC on In-hospital Mortality

Neurological symptoms were the first clinical manifestation of IE in 44.6% of patients with preoperative SNC diagnosis, and <25% of those patients were receiving antibiotic therapy at that point. Only eight patients received active antibiotic treatment in the 3 days before the neurological event, and 83% of the patients received no antibiotic treatment before SNC. The risk factors related to the presence of preoperative SNC are shown in Table 3. The median time between preoperative SNC and surgery was 18 days (range: 4–42) in patients with hemorrhagic lesions and 15 days (range: 1–44) in patients with preoperative ischemic injury (*P* = 0.433), and mortality was 16.6 and 36.6%, respectively (*P* = 0.206). About 25% of survival patients who underwent surgery within the first week (eight patients) suffered a postoperative hemorrhagic transformation compared to 12% of those who underwent surgery after the first week (25 patients) (*P* = 0.372). There was a non-significant trend to increased mortality in nine patients who underwent surgery within 7 days of presenting with SNC (55.5%) compared to those 38 patients who underwent surgery more than 7 days after SNC (38 patients) (33.3%; *P* = 0.171), but the combined risk of mortality or postoperative hemorrhagic transformation increased when surgery was required in the first week after preoperative SNC: 77.5% vs. 25% (*P* = 0.017).

**Table 3:** Risk factors for preoperative SNC.

**Risk factor**	**HR**	**95% CI**	***p***
Other than neurological embolism	8.1	3.4–19	<0.0001
Antibiotic treatment before SNC <7 days	9.1	3.3–23.9	<0.0001
Vegetation diameter >30 mm	6.4	1.7–24.4	0.006
Time to admission <10 days	2.5	1.1–6	0.029
Preoperative chronic steroid treatment	3.7	1.2–10.7	0.015
Peripheral vascular disease	4.2	0.8–21.6	0.078
Female sex	2.3	0.8–6.3	0.081
Preoperative anticoagulant treatment	2.2	0.8–5.5	0.089

*P < 0.05 is considered significant. SNC, symptomatic neurological complication; HR, hazard ratio; CI, confidence interval*.

### Effect of Preoperative SNC on Mortality During Follow-Up

**Figure 2:**
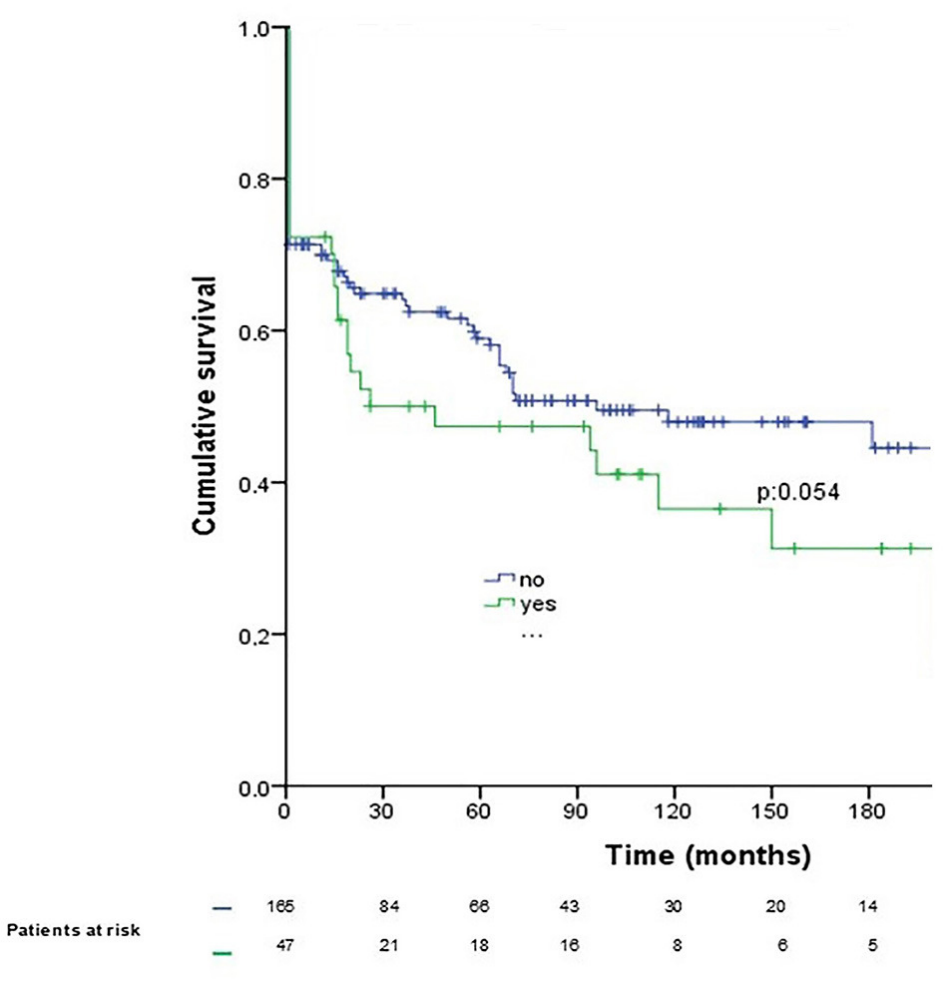
Patient survival after diagnosis with preoperative symptomatic neurological complication secondary to active left-sided infective endocarditis requiring early surgery (operative mortality included). Blue line, patients without preoperative SNC; green line, patients with preoperative SNC.

**Figure 3:**
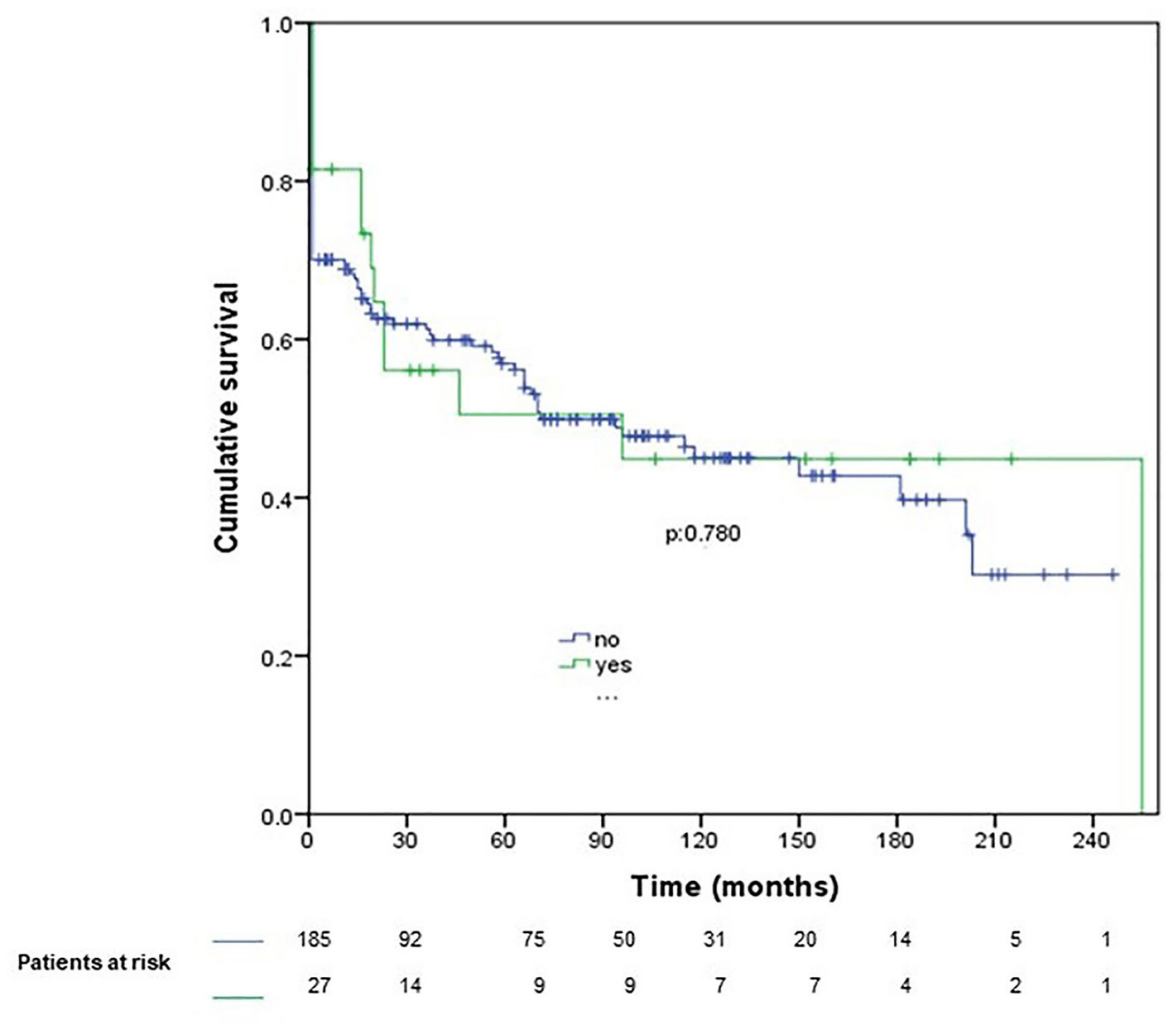
Survival after postoperative symptomatic neurological complication (operative mortality included). Blue line, patients without postoperative SNC; green line, patients with postoperative SNC.

When preoperative SNC occurred, medium-term and long-term survivals were not significantly lower (Figure 2). No differences were observed between patients with postoperative SNC and the rest of the study sample (Figure 3).

### Additional Risk Factors for Mortality and Postoperative SNC

The mortality risk factors identified in the multivariate analysis are detailed in Table 4. A new postoperative SNC was observed in only 27 (12.7%) patients: 14 (8.4%) in the no SNC group and 13 (27.7%) in the SNC group (*P* = 0.001). Table 5 shows the risk factors for new postoperative SNC.

**Table 4:** Risk factors for mortality.

**Risk factor**	**HR**	**95% CI**	***p***
Blood culture-negative IE	2.7	1.1–6.7	0.022
Annular valve abscess	2.6	1.3–5.4	0.007
Preoperative acute kidney injury	4.1	1.6–10.1	0.002
Methicillin resistant infection	7.5	2.1–26.7	0.002
Fungal endocarditis	7.1	0.9–52.8	0.055
Age >68 years	2.1	1.04–4.3	0.037
Peripheral vascular disease	7.1	1.6–31.7	0.010
Antibiotic treatment >7 days	0.2	0.1–0.6	0.001
Preoperative hemoglobin >9.6 g/dl	0.3	0.1–0.7	0.007

*P <0.05 is considered significant. HR, hazard ratio; CI, confidence interval; IE, infective endocarditis*.

**Table 5:** Risk factors for postoperative SNC in all patients and in patients without preoperative SNC.

**Risk factor**	**HR**	**95% CI**	***p***
***All patients (n = 212)***			
Preoperative SNC	2.8	1.0–7.2	0.033
*Staphylococcus aureus* IE	2.8	1.1–7	0.020
Acute renal injury	3.01	1.1–8.2	0.031
Systemic embolism	2.5	1–6.6	0.050
***Without preoperative SNC (n = 165)***			
Coronary disease	7.02	1.5–32.3	0.013
Calcium channel blockers	6.2	1.5–24.3	0.008
Valve annular abscess	3.1	0.9–10.8	0.067
Other than neurological embolism	3.06	0.8–10.4	0.074

*P < 0.05 is considered significant. IE, infective endocarditis; SNC, symptomatic neurological complication; HR, hazard ratio; CI, confidence interval*.

### Predictors of Mortality and SNC During Follow-Up

A total of 45 of 146 surviving patients (30.8%), died during follow-up period (28.8% due to sepsis, 22.2% due to cardiac failure, 17.7% due to SNC, 15.5% due to pneumonia, 6.6% due to cancer, and 8.8% due to other causes). About 57.7% required hospital readmission with sepsis (21.1%) being the most frequent cause. Survival at 1, 5, 10, 15, and 20 years was 70.5, 56.1, 45.0, 43.4, and 33.2%, respectively. Actuarial survival of patients with ALSIE requiring early surgery is shown in Figure 2. The following were identified as risk factors for mortality during follow-up using Cox regression multivariate analysis (presented as hazard ratios [95% CIs]): age (1.07 [1.039–1.10]; *P* = 0.0001), fungal IE (8.13 [1.47–44.79]; *P* = 0.016), diameter of IE vegetation > 30 mm (5.17 [2.21–12.08]; *P* = 0.0001), preoperative anticoagulant therapy (2.26 [1.16–4.40]; *P* = 0.016), and new episode of IE (3.64 [1.47–8.96]; *P* = 0.002).

Thirteen patients presented with a new IE episode: in 61.5% of cases, it was a recurrence, and in 76.9% of cases, it was due to early prosthetic valve IE. The mortality in patients diagnosed with a new IE episode was 69.2%, and in 46.2% of the cases, new-onset SNC was identified. Six of the patients re-admitted to the hospital with a new IE episode had preoperative SNC during the first IE episode, and five of them presented with ischemic lesions. The recurrence of IE (5.0 [1.1–21.5]; *P* = 0.031), *Staphylococcus aureus* IE (5.1 [1.3–18.9]; *P* = 0.014), early prosthetic valve IE (4.4 [1.0–19.5]; *P* = 0.048), diabetes mellitus (5.3 [1.3–21.4]; *P* = 0.019), and chronic steroid treatment (3.5 [1.0–12.7]; *P* = 0.049) are risk factors for new SNC during follow-up (presented as hazard ratios [95% CIs]).

## Discussion

The risk of postoperative SNC increased in patients with preoperative SNC, but we have not been able to demonstrate that preoperative nor postoperative SNC predicted a reduced immediate, medium-term, or long-term survival in the analyzed population. Mortality is higher in patients who underwent surgery within 7 days of SNC, but mortality of early surgery is acceptable after the first week of preoperative ischemic or hemorrhagic complication. Mortality found in this study in patients with ALSIE is consistent with previous reports (1–6). Lower mortalities are reported in series that exclude patients with preoperative cardiogenic shock, hemorrhagic neurological complication, or without antibiotic treatment at the time of surgery (13). In the analyzed sample, patient survival depended on the pathogen virulence (14) and factors inherent to the patient: age, associated comorbidities, and preoperative clinical status. Operative mortality almost tripled among patients with negative blood cultures, while treatment with bactericidal antibiotics for at least 7 days before surgery reduced mortality by four times.

No significant differences in mortality attributable to SNC were found, despite that these patients more frequently required early surgery (i.e., before completing seven days of antibiotic treatment). For patients with IE, consensus guidelines recommend delaying surgery until 2 weeks after an ischemic event and 4 weeks after hemorrhagic damage (9, 15), but some researchers have not observed the benefit in delaying surgery beyond 2 weeks to prevent hemorrhagic transformation or postoperative neurological deterioration after intracranial hemorrhage (16, 17). In the analyzed sample, the prevalence of hemorrhagic damage is higher than in other reports (5, 6, 18, 19), but we did not observe higher mortality in patients with hemorrhagic damage than in patients with ischemic injury. However, we did observe an increased combined risk of mortality and/or hemorrhagic transformation when surgery was required within the first week of diagnosis with SNC. This would suggest that every effort should be made to delay surgery in patients with ALSIE within the first week of diagnosis with SNC.

Neurological symptoms were the first clinical manifestation of IE in almost half of the patients with preoperative SNC diagnosis, and less than a quarter were receiving antibiotic therapy at that point. We have identified a group of patients with ALSIE with a particularly aggressive disease progression: those requiring hospitalization within 10 days of IE symptom onset, in whom the probability of SNC is 2.5 times greater. Heart failure is the most frequent cause of early surgery in this group, as well as in patients with a more favorable disease course (70% vs. 64.7%, respectively; *P* = 0.67), but the median number of days from the onset of symptoms to surgery was significantly lower (18 vs. 43; *P* < 0.0001).

The risk factors for postoperative SNC in our sample were generally related to previous embolic episodes, a preoperative symptomatic low cardiac output, and a worse prognosis of IE measured by a rapid disease progression as seen in other studies (4). However, the analysis of medium- and long-term survival of patients with preoperative and postoperative SNC did not show significantly higher mortality than those without SNC. However, we cannot absolutely exclude that the prognosis is worse in a larger sample of patients. During follow-up, IE recurrence was the main risk factor for mortality and new SNC in patients with ALSIE who had undergone early surgery.

## Limitations

These results might not be extrapolated to larger populations with different demographic and clinical characteristics. Selection bias is possible due to the low frequency of surgery in patients with SNC secondary to IE in Spain (6). Besides, the reason for severe complications that required surgical treatment would appear at different time intervals for each patient, regardless of whether they have a previous SNC and how long it has evolved. There is no doubt that all the patients included in the study meet the inclusion criteria for early surgery, nevertheless, possible bias secondary to the elapsed time between SNC and severe complications of IE who need early surgery is impossible to control. High mortality during surgery, due to preoperative cardiogenic shock, makes it difficult to tease out the effects of preoperative SNC. Finally, this is an observational study, so no cause-effect relationships can be determined at this point.

## Conclusions

Patients with ALSIE who need early hospital admission rapidly progress toward SNC. The combined risk of mortality or postoperative hemorrhagic transformation is higher in patients who underwent surgery within 7 days of SNC, but mortality of early surgery is acceptable after the first week of preoperative ischemic or hemorrhagic SNC. We have not been able to demonstrate that preoperative SNCs predicted a reduced immediate, medium-term, or long-term survival in the population analyzed in this study. No differences were identified in immediate, medium-term, or long-term survival after postoperative SNC. ALSIE recurrence is the main risk factor that jointly contributes to mortality and new SNC during follow-up.

## Data Availability Statement

The original contributions presented in the study are included in the article/supplementary material, further inquiries can be directed to the corresponding author/s.

## Ethics Statement

The study protocol (identification number PI19-1236) received full approval by both the local Institutional Research Review Committee and the Clinical Research Ethics Committee of Hospital Clínico Universitario de Valladolid. Avda. Ramón y Cajal 3. 47003 Valladolid Spain. The patients/participants provided their written informed consent to participate in this study.

## Author Contributions

YC and ÁG have contributed to the design, planning, conduct, and reporting of the work described in the article. BS and EV have contributed to the conduct and reporting of the work described in this article. All authors have finally approved the manuscript.

## Conflict of Interest

The authors declare that the research was conducted in the absence of any commercial or financial relationships that could be construed as a potential conflict of interest.

## Publisher's Note

All claims expressed in this article are solely those of the authors and do not necessarily represent those of their affiliated organizations, or those of the publisher, the editors and the reviewers. Any product that may be evaluated in this article, or claim that may be made by its manufacturer, is not guaranteed or endorsed by the publisher.
